# Effects of weak transcranial alternating current stimulation on brain activity—a review of known mechanisms from animal studies

**DOI:** 10.3389/fnhum.2013.00687

**Published:** 2013-10-23

**Authors:** Davide Reato, Asif Rahman, Marom Bikson, Lucas C. Parra

**Affiliations:** Department of Biomedical Engineering, The City College of The City University of New YorkNew York, USA

**Keywords:** transcranial alternating current stimulation (tACS), oscillations, animal models, slow wave, gamma, electroencephalogram (EEG), entrainment, transcranial direct current stimulation (tDCS)

## Abstract

Rhythmic neuronal activity is ubiquitous in the human brain. These rhythms originate from a variety of different network mechanisms, which give rise to a wide-ranging spectrum of oscillation frequencies. In the last few years an increasing number of clinical research studies have explored transcranial alternating current stimulation (tACS) with weak current as a tool for affecting brain function. The premise of these interventions is that tACS will interact with ongoing brain oscillations. However, the exact mechanisms by which weak currents could affect neuronal oscillations at different frequency bands are not well known and this, in turn, limits the rational optimization of human experiments. Here we review the available *in vitro* and *in vivo* animal studies that attempt to provide mechanistic explanations. The findings can be summarized into a few generic principles, such as periodic modulation of excitability, shifts in spike timing, modulation of firing rate, and shifts in the balance of excitation and inhibition. These effects result from weak but simultaneous polarization of a large number of neurons. Whether this can lead to an entrainment or a modulation of brain oscillations, or whether AC currents have no effect at all, depends entirely on the specific dynamic that gives rise to the different brain rhythms, as discussed here for slow wave oscillations (∼1 Hz) and gamma oscillations (∼30 Hz). We conclude with suggestions for further experiments to investigate the role of AC stimulation for other physiologically relevant brain rhythms.

## Oscillations in the brain and tACS

Oscillations are ubiquitous in the human brain, ranging from ultra-slow (0.05 Hz) to ultra-fast oscillations (500 Hz) (Ward, 2003; Buzsaki and Draguhn, 2004). These oscillations occur in the brain during different behavioral states and their power (amplitude) is commonly modulated during cognitive/behavioral tasks (Buzsaki and Wang, 2012). Many oscillatory rhythms are usually simultaneously present and can modulate each other (e.g., fast-oscillations vary in amplitude with the phase of the slower rhythm, and this has been hypothesized to be relevant i.e., during sensory selection) (Schroeder and Lakatos, 2009). Abnormal brain rhythms have also been shown to correlate with pathological conditions, like Parkinson’s disease, Alzheimer’s and epileptic seizures (Brown et al., 2001; Worrell et al., 2004; Montez et al., 2009). Therefore, a number of research studies are based on the assumption that modulating brain rhythms has the potential to affect cognitive performance and may be used to treat neurological disorders. This is particularly true for interventions using transcranial alternating current stimulation (tACS), which target specific brain oscillations (Antal et al., 2008; Pogosyan et al., 2009; Moliadze et al., 2012; Brignani et al., 2013; Santarnecchi et al., 2013; Struber et al., 2013). This short review intends to: (1) elucidate the known mechanisms of AC stimulation from recent *in vitro* and *in vivo* animal findings, (2) suggest the key mechanisms that determine the effects of AC stimulation and (3) propose future animal studies that may guide further development of tACS clinical protocols. We focus exclusively on electrophysiological animal data that provide direct experimental evidence for the cellular and network mechanisms of AC stimulation effects, and review computational models of this data, where available. For a review on the effects of tACS in human studies please refer to (Antal and Paulus, 2013; Herrmann et al., 2013; Marshall and Binder, 2013).

## DC vs. AC stimulation

Transcranial electrical stimulation in humans has been predominantly applied using constant electric currents (often called direct current (DC)) to transiently modulate cognitive and behavioral function (Nitsche and Paulus, 2000; Nitsche et al., 2005; Stagg and Nitsche, 2011). The changes in neural excitability leading to the modulation of brain function have been extensively studied under controlled situations using animal models of DC stimulation, both *in vivo* and *in vitro* (Bindman et al., 1964; Purpura and Mcmurtry, 1965; Chan et al., 1988; Bikson et al., 2004; Rahman et al., 2013). During subthreshold DC stimulation, the current flowing in the brain modulates the cellular excitability of resting neurons by changing the membrane voltage (transmembrane polarization). The induced polarization of the soma is at most 0.2 mV for each 1 V/m of applied field (Bikson et al., 2004; Radman et al., 2009). Given that conventional protocols (1 mA stimulation intensity) produce electric fields of 1 V/m maximum (Datta et al., 2009), the expected maximum polarization is only 0.2 mV at pyramidal somas. This resulting somatic polarization, albeit small, can affect firing rate for a large number of neurons (Fröhlich and McCormick, 2010; Reato et al., 2010).

Recently, an increasing number of human studies have employed time-varying current stimulation to influence cortical excitability (Marshall et al., 2006; Antal et al., 2008; Kirov et al., 2009; Kar and Krekelberg, 2012; Moliadze et al., 2012; Brignani et al., 2013). tACS refers to electrical stimulation where current is not constant (DC) but alternates between the anode and the cathode (switching polarity) with a sinusoidal waveform. Clinically, tACS may be applied using a wide range of stimulation frequencies and intensities, including with a DC offset (Marshall et al., 2006). While it has been shown that tACS can modulate cortical excitability, electroencephalogram (EEG) oscillations, and cognitive processes (for review see Antal and Paulus, 2013; Herrmann et al., 2013; Marshall and Binder, 2013), there is also evidence for a failure to produce such effects under some circumstances (Brignani et al., 2013). The predominant hypothesis of tACS action is that alternating fields can increase or decrease power of oscillatory rhythms in the brain in a frequency-dependent manner by synchronizing or desynchronizing neuronal networks—this generic hypothesis, applied across brain regions and frequencies, warrants analysis for mechanistic feasibility.

## tACS sinusoidally modulates the neuronal membrane potential

**Figure 1:**
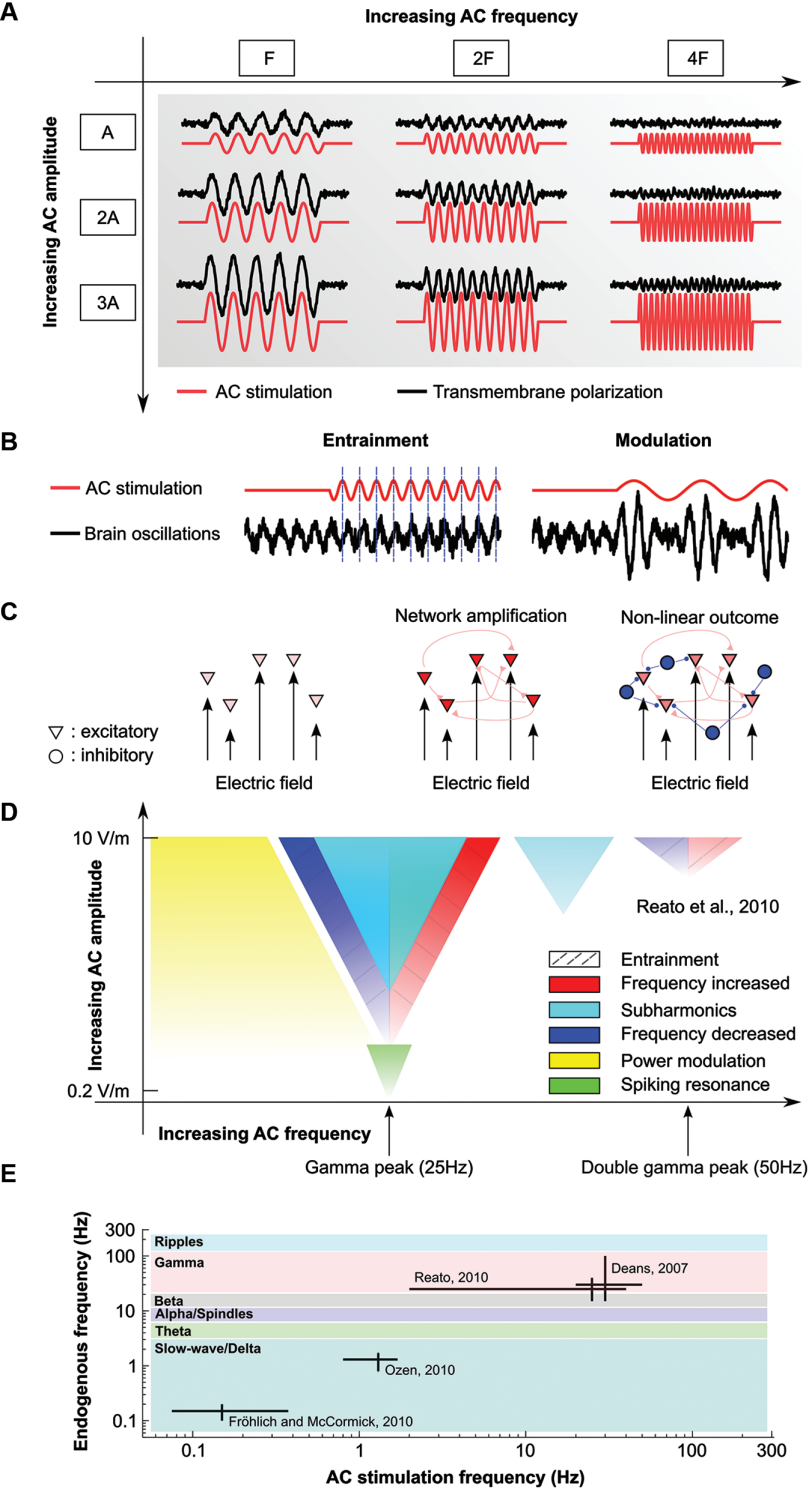
**Effects of AC stimulation on single and network of neurons**. **(A)** Schematic of the effects of AC stimulation on resting neurons. Sinusoidal electrical stimulation (AC, red lines) sinusoidally modulates the membrane voltage (black lines). The membrane polarization increases with increasing stimulation amplitude (A, 2A, 3A) but decreases with increasing stimulation frequency (F, 2F, 4F). **(B)** Examples of possible effects of weak AC stimulation on oscillations. AC stimulation can entrain the oscillations by shifting their phase (*left*) or modulate its power at the stimulation frequency (*right*). **(C)** Schematics of the effects of AC stimulation on network of neurons. While the effects of electrical stimulation on single neurons are small (*left*), synaptically connected neurons can provide feedback that amplifies the effects of stimulation (*center*). However, active neurons create network oscillations, usually set by the level of activity of excitatory and inhibitory neurons (triangle and circles, respectively). In this case, the effects of stimulation cannot *a priori* always be determined (*right*). **(D)** Summary of the known effects of weak AC stimulation on gamma oscillations. AC fields can entrain spiking activity at very low intensities (green), while low-frequency stimulation modulates the power of gamma oscillations (yellow). Gamma oscillations can be entrained (dashed lines) by using frequencies close to the endogenous one or double that frequency (increase in frequency, red, or decrease in frequency, blue). Frequencies close to the stimulation frequency but higher intensity induce pacing at half of the stimulation frequency (cyan). Color gradients indicate size of the effects. Frequencies that do not match the endogenous frequency, for example, can still affect the oscillations if the stimulation amplitude is increased. The figure is adapted from Reato et al. (2010). **(E)** Schematics of the *in vitro* and *in vivo* animal studies applying AC (sinusoidal) stimulation on oscillatory rhythms. The main frequency of the endogenous oscillations of interest (vertical axis) and the stimulation frequencies applied in the different studies (horizontal axis) demonstrate the limited range of neural rhythms that have been described in the animal literature. Colors indicate frequency bands. Also note the log-scale axes.

At the cellular level sinusoidal AC electric fields applied extracellularly, when directed across pyramidal neurons (along the somatodendritic axis), will sinusoidally alter the transmembrane potential (Chan and Nicholson, 1986). Neurons polarize proportionally with field intensity during AC (Deans et al., 2007) and DC stimulation (Bikson et al., 2004; Fröhlich and McCormick, 2010) with no obvious lower threshold. Small polarization of the membrane can then lead to modulation of firing rate for active neurons (Chan and Nicholson, 1986; Ozen et al., 2010; Reato et al., 2010). However, as first reported in Deans et al. (2007) the cell susceptibility to polarization (mV of polarization per V/m applied electric field) of hippocampal pyramidal neurons (in CA3 is approximately inversely proportional to the applied field frequency (DC: 0.18 mV per V/m, 50 Hz AC: 0.07 mV per V/m) indicating a decrease in membrane response to increasing stimulation frequency. The efficacy of AC field frequency derives from the passive properties of biological membranes (that acts like a low-pass filter with a time constant of 5–20 ms, Figure 1A). While the effects of AC fields on single neurons are then expected to be smaller than the effects of DC fields, there are a few studies indicating that networks of neurons can exhibit a higher sensitivity to AC fields.

## AC stimulation can entrain neuronal oscillations

Kainic acid in rat hippocampal slices can induce high-beta/gamma oscillations (15 Hz–100 Hz) in the CA3 region. Deans et al. (2007) showed that the frequency and power of these gamma oscillations were modulated by the application of AC fields (< 8 V/m, at 20 or 50 Hz). More specifically, the authors showed that AC fields shift the original peak frequency of the oscillations to the stimulation frequency or a subharmonic (f/2, f/3…) of the stimulation. Fields as low as 0.25 V/m (peak amplitude) were able to modulate the oscillations in 20% of slices, while 0.5 V/m modulated gamma rhythms in 50% of slices (the effects were amplitude-dependent). These results provide evidence of an important effect of AC stimulation, specifically, entrainment of gamma oscillations to the applied field (Figure 1B). Note that while the frequency of the oscillatory rhythm spanned across two different physiological bands (high beta/gamma), the mechanisms that generate oscillations are probably the same. Previous studies (for a review see Bartos et al., 2007) suggest that kainic acid induce gamma-like oscillations (not beta).

Other experiments have shown that gamma oscillations in cell cultures can be driven electrically if frequency is carefully adjusted (Fujisawa et al., 2004). Perfusion of brain slices with high potassium solution can induce bursting firing that can also be entrained with very weak pulsed stimulation (0.3 V/m peak amplitude, Francis et al., 2003). Low-intensity pulsed stimulation can also modulate spike and wave seizures (Berenyi et al., 2012).

Entrainment of ongoing neuronal activity to AC stimulation at frequencies mimicking the frequency of cortical slow oscillations (0.8–1.7 Hz) was demonstrated across multiple cortical areas by Ozen et al. (2010) in anesthetized rats. Ozen et al. (2010) reported that membrane potential and unit activity were modulated by AC stimulation in cortical and hippocampal areas (20% and 16% of units were entrained in cortex and hippocampus, respectively). Postmortem calibration suggested that 1 V/m in the extracellular space was sufficient to phase-lock units. Intracellular recordings indicated that the intensities that effectively phase-locked induced 2–3 mV polarization. Increasing stimulation intensity recruited an increasingly larger population of spiking neurons without an evident threshold for this effect, consistent with previous *in vitro* work. Importantly, the modulation with applied AC fields was more effective when the brain endogenously exhibited slow-wave oscillations suggesting a form of resonance.

## Neuronal networks can amplify the effects of AC stimulation

Additional support for the hypothesis that active networks could be more sensitive than single cell to electric fields was presented for slow-wave oscillations in ferret cortical slices (Fröhlich and McCormick, 2010). When perfused with *in vivo*-like artificial cerebro-spinal fluid (ACSF) ferret slices exhibit such slow-wave oscillations. During DC stimulation in the range of 0.5–4.0 V/m, this study reported a higher frequency of these slow oscillations and determined that this effect was due to a reduced duration of the DOWN state (see below). AC stimulation entrained slow wave oscillations in an amplitude and frequency-dependent manner. In particular, AC fields with frequency matched to the endogenous oscillation led to more periodic up states at a rate that matched the stimulus frequency. By combining these experimental findings with a computational model of slow-wave oscillations, Fröhlich and McCormick (2010) showed that these effects could be explained by the weak polarization of the membrane acting simultaneously on many synaptically connected neurons (Figure 1C). Recently, the same group has shown frequency-specific entrainment of multi-unity activity also *in vivo* (Ali et al., 2013).

In a previous study, we analyzed the effects of weak electrical stimulation on carbachol-induced gamma oscillations in the CA3 region of rat hippocampal slices (Reato et al., 2010). The frequency of the stimulation was varied from constant DC to 40 Hz AC stimulation with effects observed at field-intensities as low as 0.2 V/m. DC stimulation modulated the power of the oscillations, with soma-depolarizing fields increasing the power and soma-hyperpolarizing field decreasing it. The effects of AC stimulation varied qualitatively for different frequencies. Low-frequency AC stimulation (2–7 Hz) strongly modulated the gamma power at the stimulation frequency (Figure 1B). Interestingly, at stimulation frequency close to the endogenous rhythm, we saw an increase of power at half of the stimulation frequency (sub-harmonic). Moreover, very low-amplitude stimulation (0.2 V/m) entrained firing activity only when the frequency of the endogenous rhythm was matched by the stimulation (resonance). Despite the complexity of the experimental results, we were able to fully account for all the measured effects with a computational model (Figure 1D). The model suggested that the effects derived from modulation of firing rate, spike timing and the balance between excitation and inhibition. Recurrent feedback between excitatory and inhibitory neurons leads to a compensatory increase of inhibition whenever excitation is increased, e.g., by field-induced depolarization. Such balanced networks are often invoked to explain normal physiological rhythms (Shu et al., 2003; Haider et al., 2006; Atallah and Scanziani, 2009). Although inhibitory neurons are less sensitive to extracellular electrical stimulation because of their symmetric geometry (Radman et al., 2009), it is also evident that since inhibitory neurons are in general more depolarized, they are likely more sensitive to small voltage fluctuations than excitatory neurons. Thus, even assuming no direct polarization of inhibitory neurons, networks in the brain are always active, and modulation of excitatory neurons may affect (indirectly) inhibitory neurons leading to non-trivial effects (Moliadze et al., 2012; Krause et al., 2013). Importantly, our study showed that the effects of AC stimulation cannot be reduced to a simple increase or decrease in power or frequency of the network oscillations (Reato et al., 2010).

## The effects of AC stimulation depends on brain activity

The combined results from the previous studies suggest some general principles regulating the effects of AC stimulation on network of neurons. AC stimulation of inactive neurons induces a simple sinusoidal modulation of neuronal membrane voltage that exhibits low-pass filtering properties. In the context of tACS, this would lead to the conclusion that high frequency stimulation (hundreds of hertz) may be ineffective in modulating brain activity (almost zero induced polarization, Figure 1A). However, the studies discussed above showed that network activity and the coherent stimulation of many neurons can amplify the effects of otherwise very small membrane polarizations. AC fields can modulate rate and timing of spiking neurons (Chan and Nicholson, 1986; Fröhlich and McCormick, 2010; Reato et al., 2010) and thus modulate recurrent interaction between neurons. Neurons whose activity is modulated by electrical stimulation will in turn modulate the activity of other neurons, generating a feedback loop that can amplify the effects of stimulation on single neurons (Figure 1C). When neurons are in an excitable state, oscillatory rhythms can emerge. Such networks are often characterized by a tight dynamical balance between excitation and inhibition that determines the firing rate and timing of excitatory and inhibitory neurons. When altering firing rate or timing in some neurons the network can “react” to compensate or magnify the effects in a non-trivial way.

For instance, as one might expect, AC fields can entrain network oscillations when stimulating with the frequency of the networks’ own rhythm. This was observed for a variety of preparations (Francis et al., 2003; Fujisawa et al., 2004; Deans et al., 2007; Fröhlich and McCormick, 2010; Reato et al., 2010). Yet, in some circumstances the network is paced at half of the stimulation frequency instead. When the stimulation frequency is much lower than the network rhythm some oscillations remain unaffected (slow waves) while others are strongly modulated in power (gamma, Figure 1B). Even a form of stochastic resonance was observed in high-potassium slice preparation with a combination of AC and random noise stimulation (Gluckman et al., 1996).

These results suggest that the effects of AC stimulation are not always readily predicted and are certainly not a simple modulation on a one-dimensional scale of oscillation power (Figure 1D) as often assumed.

A key factor to consider when trying to understand and anticipate the effects of AC stimulation are the exact mechanisms underlying different endogenous rhythms. For example, slow-wave oscillations (0.5–4 Hz), typical oscillatory activity during sleep (Sejnowski and Destexhe, 2000; Huber et al., 2004), represent a succession of active states of neurons (UP state), characterized by high spiking activity and strong synaptic interaction with inactive states (DOWN states) with almost no firing (Steriade et al., 1993). The high level of activity of the UP state depletes the cellular resources and the self-sustained excitatory activity collapses (think of a group of children playing past their bed-time) thus transitioning to the DOWN state. This transition follows its internal dynamic and cannot be readily modulated. On the other hand, the transition from DOWN to UP state can be driven by even a single spike. Essentially the cellular resources have recovered during the quiescent phase and the network is ready to start up again. A small “kick” can get the avalanche of activity up again (the first kid waking up will get the group going again). Naturally, this DOWN-UP transition could be easily driven by any well-timed external stimulus. The results of Fröhlich and McCormick (2010) indeed suggest that neurons can be quickly driven with small polarization to the UP state. AC stimulation can then more easily entrain oscillations when the stimulus frequency matches the endogenous frequency by shortening the DOWN states (Reato et al., 2013).

In contrast, gamma oscillations are generated by the interplay of excitatory and inhibitory neurons, where excitatory neurons provide the excitation necessary for inhibitory neurons to set the timing of the network (like a clock, Fisahn et al., 1998; Bartos et al., 2007). The generation of half-harmonics when stimulating with the endogenous frequency results from increased temporal alignment of firing of excitatory neurons; this increased synchrony causes a stronger excitatory volley to inhibitory neurons which are thus more strongly activated forcing the network to suppress the next “beat”—thus the network “skips a beat” resulting in half as many cycles, i.e., half harmonic (Reato et al., 2010). The strong modulation of gamma oscillations with slow AC stimulation is a result of an overshoot of the dynamic balance between excitation and inhibition, akin to periodically hitting the break in a standing car with automatic drive.

In summary, the effects of stimulation cannot be established *a priori* without understanding the specific mechanisms underlying neuronal network dynamic.

## Future directions

tACS is currently being explored as a tool to modulate brain rhythms in a number of human experiments. We have reviewed here the few *in vitro* and *in vivo* studies of mechanisms underlying the effects of tACS. These studies have shown that active networks are very sensitive to electrical stimulation and in particular to AC stimulation. The effects are mediated by a small polarization of the neuronal membrane potential that lead to changes in spike rate and timing, which are then magnified by the network dynamic.

 However, the effects of stimulation depend strongly on the specific network dynamics and in this sense there are still wide gaps in our understanding of AC stimulation. Specifically, alpha (8–12 Hz), beta (13–30 Hz) and theta (4–7 Hz) oscillations as well as spindle activity and sharp wave ripples are all important physiological oscillatory rhythms that have been extensively linked to cognitive phenomena such as attention, motor control, memory retrieval and memory consolidation. Yet, there is no electrophysiological data from cellular or network level studies on the effects of weak electric stimulation on any of these rhythms (Figure 1E). Several tACS studies involved stimulation using alpha/beta frequencies (Kanai et al., 2008; Pogosyan et al., 2009; Zaehle et al., 2010; Feurra et al., 2011; Neuling et al., 2012) or combining different stimulation frequencies, including theta (4–7 Hz) and beta (13–30 Hz) over the primary motor cortex (Schutter and Hortensius, 2011). The hope is to facilitate endogenous oscillations at these frequencies, but the lack of experimental evidence for this makes the study of the cellular and network effects of stimulation on these rhythms particularly important.There are *in vitro* preparations that generate beta rhythms (Shimono et al., 2000) and thalamo-cortical spindles (Von Krosigk et al., 1993; Tancredi et al., 2000), while to our knowledge, there are currently no *in vitro* models of alpha oscillations. Theta oscillations (Cappaert et al., 2009; Goutagny et al., 2009) and ripples (Behrens et al., 2005; Nimmrich et al., 2005) can also be pharmacologically induced. Rodent brain slice preparations are obviously a poor model for human brain rhythms; nevertheless they have proven to be a useful tool to study the cellular substrate of tACS particularly because stimulation may be applied in a controlled setting and specific interactions between networks of oscillating neurons can be systematically probed. We also note that transcranial stimulation in humans is thought to generate weaker fields (< 1 V/m) than what is used in slices models (= 1 V/m), combined with cortical folding which leads to uncontrolled field orientations, it is hard to predict the outcome of any one human experiment based on slice experiments alone. Animal *in vivo* experiments offer a more physiological environment to test stimulation effects. However, presently they offer limited control over the direction and intensity of current flow; a technical limitation that can be addressed through quantitative models of current flow in animals (Datta et al., 2009; Marquez-Ruiz et al., 2012).

The results shown here suggest that tACS may go beyond frequency coupling of the stimulation and endogenous activity (for instance alpha-modulated alpha) and could perhaps be used for cross frequency coupling. For example, recent studies have demonstrated that low-frequency oscillations can modulate the amplitude of higher frequency oscillations (like theta-modulated gamma) and that the effects are functionally and behaviorally relevant, including for working and spatial memory. However, to our knowledge, stimulation of brain rhythms with lower frequencies to modulate the amplitude of higher frequency oscillations has not been explored yet in human brain stimulation. In Reato et al. (2010) we demonstrated that gamma oscillations were strongly modulated by lower-frequency stimulation (including theta frequencies), raising the possibility that such modulation may also be possible in humans. Practically, cross frequency coupling of stimulation and endogenous activity has the additional technical benefit that tACS would not produce stimulation artifacts at the frequency of interest (Neuling et al., 2012).

The *in vivo* and *in vitro* studies reviewed here provide evidence for the acute effects of stimulation on gamma and slow-wave oscillations. Yet, it is important to note that none of the animal studies reviewed above report lasting effects, i.e., as soon as the AC fields are turned off, the observed effects seemingly disappear. Admittedly, these studies did not apply long-duration stimulation (minutes) and thus long-term effects were not expected or noted. Clearly, long-term effects at the cellular level must mediate the long-term effects observed in human studies, thus, there is an urgent need to clarify the underlying mechanisms. Additionally, stimulation dosage including duration, intensity (Moliadze et al., 2012; Neuling et al., 2013), frequency (Zaehle et al., 2010; Struber et al., 2013), and electrode montage may interact synergistically to influence the post-stimulation effects. For DC stimulation, several studies have shown long-lasting synaptic effects after stimulation *in vitro* and *in vivo* (Bindman et al., 1962; Gartside, 1968a,b; Fritsch et al., 2010; Ranieri et al., 2012). Specifically, Brain-derived neurotrophic factor (BDNF; Fritsch et al., 2010), adenosine (Marquez-Ruiz et al., 2012), N-methyl-D-aspartate receptor (NMDA-receptors; Liebetanz et al., 2002), regulation of gene expression (Ranieri et al., 2012), and protein synthesis (Gartside, 1968b) have all been implicated in synaptic changes induced by weak DC stimulation. However, it’s unclear how DC-induced long-term plasticity relates to AC stimulation. Bawin et al. (1984) have shown that AC stimulation can induce lasting effects on evoked responses (Bawin et al., 1984), but these results could not be readily reproduced (Deans et al., 2007). The *in vitro* preparations we cited in this review may be a good tool to test the effects of long-duration AC stimulation on neuronal networks. Understanding the acute and long-lasting effects of tACS at the cellular and network level along with physiological insights from human experiments may help to rationally design clinical stimulation protocols that aim to augment cognitive and behavioral function.

## Conflict of interest statement

The authors declare that the research was conducted in the absence of any commercial or financial relationships that could be construed as a potential conflict of interest.
